# Sand fly population dynamics in areas of American cutaneous leishmaniasis, Municipality of Paraty, Rio de Janeiro, Brazil

**DOI:** 10.1038/s41598-022-20702-w

**Published:** 2023-03-03

**Authors:** Vanessa Rendeiro Vieira, Gustavo Marins de Aguiar, Alfredo Carlos Rodrigues de Azevedo, Elizabeth Ferreira Rangel, Anthony Érico Guimarães

**Affiliations:** 1 Laboratório Interdisciplinar em Vigilância Entomológica de Diptera e Hemiptera, Instituto Oswaldo Cruz/FIOCRUZ, Rio de Janeiro, RJ Brazil; 2 Laboratório de Diptera, Instituto Oswaldo Cruz/FIOCRUZ, Rio de Janeiro, RJ Brazil

**Keywords:** Developmental biology, Ecology, Zoology

## Abstract

The present study is the second and last part of the study that investigated the fauna and behavior of sand flies in areas prone to cutaneous leishmaniasis outbreaks, in the State of Rio de Janeiro, in the municipality of Paraty. To collect the sand flies, CDC and Shannon light traps were used, installed in the peridomiciliary and forest areas, and manual suction tubes on the walls of the home and in the animal shelters. A total of 102,937 sand flies, belonging to nine genera and 23 species were captured from October 2009 to September 2012. Regarding the monthly frequency of sand flies, the period of highest density was from November to March, with a maximum peak in January. The lowest density was observed in June and July. In the studied area, the species of epidemiological importance, *Nyssomyia intermedia*, *Pintomyia fischeri*, *Migonemyia migonei* and *Nyssomyia whitmani*, were found in all months of the year, a period in which residents may be in contact with these vectors of the etiological agent of cutaneous leishmaniasis.

## Introduction

After the publication of the first case of Diffuse Cutaneous Leishmaniasis (LCD), a rare and severe form of the disease, caused by *Leishmania mexicana amazonensis* in the State of Rio de Janeiro, in the city of Paraty, by Azeredo-Coutinho et al.^1^, interest arose in observing the fauna and behavior of sandflies in the region and this study was divided into two parts, where in the first article was published the fauna and behavior of sand flies in the city of Paraty and examined the abundance index using the location and capture method^2^. The present and last article describes the monthly frequency of sand flies of epidemiological importance in this area.

The PAHO/WHO^3^ estimates that over 12 million people worldwide are infected by the etiological agent transmitter of leishmaniasis, with approximately 350 million people living in areas of risk for this disease. Brazil is one of four countries in the Americas, along with Colombia, Peru, and Nicaragua, where cutaneous leishmaniasis is highly concentrated.

Leishmaniasis is a parasitic disease that is considered one of the six major infectious diseases, owing to its high detection rate and ability to produce deformities. It is a public health problem in 88 countries, distributed among the continents of the Americas, Europe, Africa, and Asia, with approximately 1 to 1.5 million cases per year^4^.

In Brazil, from 2001 to 2020, approximately 465,000 cases were reported, being 10% in the Southeast region. The state of Rio de Janeiro had 6% of the cases registered in the Southeast region and, since the beginning of the last century and in the last decades, the disease was observed in epidemic outbreaks in different municipalities, including some economically developed, such as the region Metropolitan, with 43% of the cases, followed by Costa Verde with 20%. From the municipalities that compose this administrative region, Paraty concentrated the largest number of cases, 56%, followed by Angra dos Reis, 34% and Mangaratiba, with 10%^5^. In these areas, from 1991 to 2020, a large population increase was observed, especially in the municipalities of Mangaratiba, Angra dos Reis and Paraty, causing an increase in the demographic density of 49.7 inhabitants/km^2^ in the decade of 1990 to 10.3 inhabitants/km^2^ in the present day in Mangaratiba; 103.7 to 205.6 inhabitants/km^2^ in Angra dos Reis and 25.9 to 40.6 inhabitants/km^2^ in Paraty^6^.

The progressive expansion of leishmaniasis has raised serious concerns regarding the severity of the disease, with the ability to broaden its distribution related to either an extensive human intervention resulting in environmental imbalance or to changes in its epidemiological profiles. These changes enable etiological agents to adapt to new hosts and consequently be introduced to residential and peridomiciliary environments^7^.

Knowledge on various epidemiological aspects of cutaneous leishmaniasis in Rio de Janeiro still requires further investigation, in addition to the epidemiological aspects related to the parasite-vector-host interaction and the different transmission routes, especially as most reported cases are disregarded^8^.

Understanding seasonality curves, especially for species of medical and veterinarian interest, is important to aid more effective control measures. Thus, the present study aimed to relate the monthly frequency of the key sand fly species to climate variations of temperature, relative humidity, and precipitationl with the different types and places of capture.

## Material and methods

**Figure 1:**
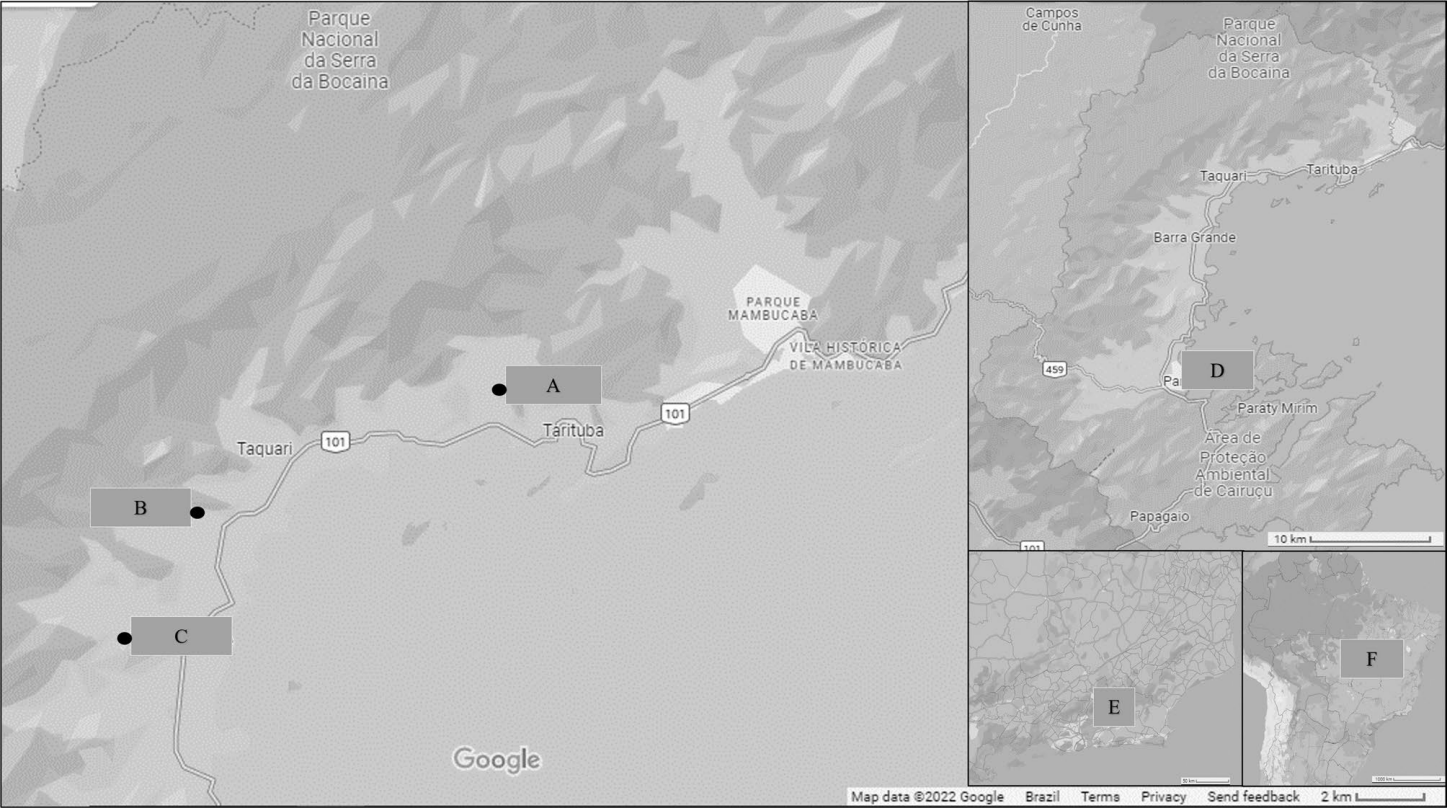
Satellite Image of the Study Area, in the neighborhoods of São Gonçalo (**A**), São Roque (**B**) and Barra Grande (**C**), Municipality of Paraty (**D**), State of Rio de Janeiro (**E**), Brazil (**F**). Map data ©2022 Google.

The municipality of Paraty is located on the southern coast of Rio de Janeiro, geographic coordinates of 23°56′26″ S, 46°19′47″ W, and is 242 km away from the city of Rio de Janeiro along Highway BR-101 (also known as Rio-Santos Highway), which links the state of Rio de Janeiro to the municipality of Santos, state of São Paulo (Fig. 1). The city is located in its entirety in the Atlantic Forest Biome and consists of several Conservation Units such as the Juatinga Ecological Reserve, within the Cairuçu Environmental Protection Area, which overlaps in several places with the Serra da Bocaina National Park and borders the Serra do Mar State Park^9–12^. The region is composed of dense ombrophilous forest (tropical rainforest) with major mountain ranges and plateaus; the climate is wet and without a dry season and has temperature averages of 17 °C minimum and 24 °C maximum, and an annual rainfall of 1,968 mm, Based on a 30-year data set^13^.

The first case of diffuse cutaneous leishmaniasis in the State of Rio de Janeiro, municipality of Paraty, reported by Azeredo-Coutinho et al.^14^ awakened interest in conducting this systematic study in the area. For this, the house where the autochthonous case occurred, in the neighborhood of São Gonçalo (-2302400 -4461051), was chosen as the capture station. Captures where performed monthly, from October 2009 to September 2011, the team stayed for four consecutive nights. Sand flies were collected using manual suction tubes on the internal and external walls of the house (simultaneously), in the annexes of domestic animals (a chicken coop, a pigsty, a corral and a kennel) and with a Shannon trap in the peridomicile, which corresponds to the area between the house and the edge of the forest, where the animal attachments and some fruit trees are found, and in the forest (simultaneously), from 6 to 10 pm. The HP model of CDC light traps^15^ were installed four traps distributed in the household, peridomicile, edge of the forest, and in the forest, about 300 m from the house (one CDC light trap in each place, about 30 cm from the ground), from 6 pm to 8 am, for four nights.

The research was extended to the neighboring districts of São Roque (-2307183 -4470358) and Barra Grande (-2309269 -4469958), as these places were frequented by the person who had the autochthonous case. The captures of sand flies were carried out from October 2011 to September 2012, monthly for four consecutive nights, using only the CDC light traps installed four traps distributed in the household, peridomicile, edge of the forest, and in the forest, about 300 m from the house (one trap in each location) from 6 pm to 8 am.

The sand flies in the manual suction tubes and the light trap, were placed at low temperature (− 4 °C) for 10 min and then transferred to glasses containing 70% alcohol, with respective labels indicating date, the collection and sorting location. In the laboratory, the sand flies of the CDC light traps were then separated from other insects, and the specimens were assembled using a stereoscopic microscope, after processing the specimens adopting the Young and Perkins^16^ technique, modified by Aguiar et al.^17^, and specific species diagnosis were made using a bacteriological microscope utilized for examining morphological characteristics, the designation of Galati^18^ was used and the proposal of abbreviations of generic and subgeneric by Marcondes^19^. The specimens were deposited in the entomological collection of the Diptera Laboratory, sector of sand fly, Oswaldo Cruz Institute, FIOCRUZ.

To analyze the data, were used the absolute frequency and Williams’ average values, according to Haddow^20,21^ and Forattini et al.^22^, to evaluate the monthly frequency of sand flies species of epidemiological importance. All the analyses were performed using Microsoft Excel (2002).

Pearson's correlation was used to observe the correlation between climate variation and the number of sandflies, this analysis varies from − 0.1 to 0.1, where − 0.7 or less indicates a strong and negative correlation and when 0.7 or more indicates a strong and positive correlation.

Climatic data (temperature, relative humidity and precipitation) from the Automatic Weather Station for Surface Observation (Parati-A619) of the National Institute of Meteorology^13^ were consulted.

## Results

A total of 102,937 sand flies were collected, belonging to nine genera and twenty-three species, *Nyssomyia intermedia* (Lutz & Neiva 1912), with 63,1% of the captured specimens, *Pintomyia fischeri* (Pinto 1926), with 23,2%, *Migonemyia migonei* (França 1920), with 11,1%, *Nyssomyia whitmani* (Antunes and Coutinho 1939), with 1,6%, and the other species added together representing only 1%, *Pintomyia monticola* (Costa Lima 1932), *Pintomyia bianchigalatiae* (Andrade Filho, Aguiar, Dias and Falcão 1999), *Psathyromyia shannoni* complex (Dyar 1929), *Pintomyia pessoai* (Coutinho and Barretto 1940), *Psychodopygus ayrozai* (Barretto and Coutinho 1940), *Evandromyia edwardsi* (Mangabeira 1941b), *Psathyromyia lanei* (Barretto & Coutinho, 1941), *Psathyromyia pascalei* (Coutinho & Barretto, 1941c), *Psathyromyia barrettoi* (Mangabeira, 1942a), *Expapillata firmatoi* (Barretto, Martins & Pellegrino, 1956), *Psychodopygus arthuri* (Fonseca, 1936), *Psychodopygus lloydi* (Antunes, 1937), *Brumptomyia guimaraesi* (Coutinho & Barretto, 1941), *Psychodopygus geniculatus* (Mangabeira, 1941c), *Micropygomyia schreiberi* (Martins, Falcão and Silva 1955), *Psathyromyia aragaoi* (Costa Lima, 1932), *Brumptomyia avellari* (Costa Lima, 1932), *Micropygomyia quinquefer* (Dyar, 1929) and *Brumptomyia cardosoi* (Barretto & Coutinho, 1941a) in order of frequency.

**Figure 2:**
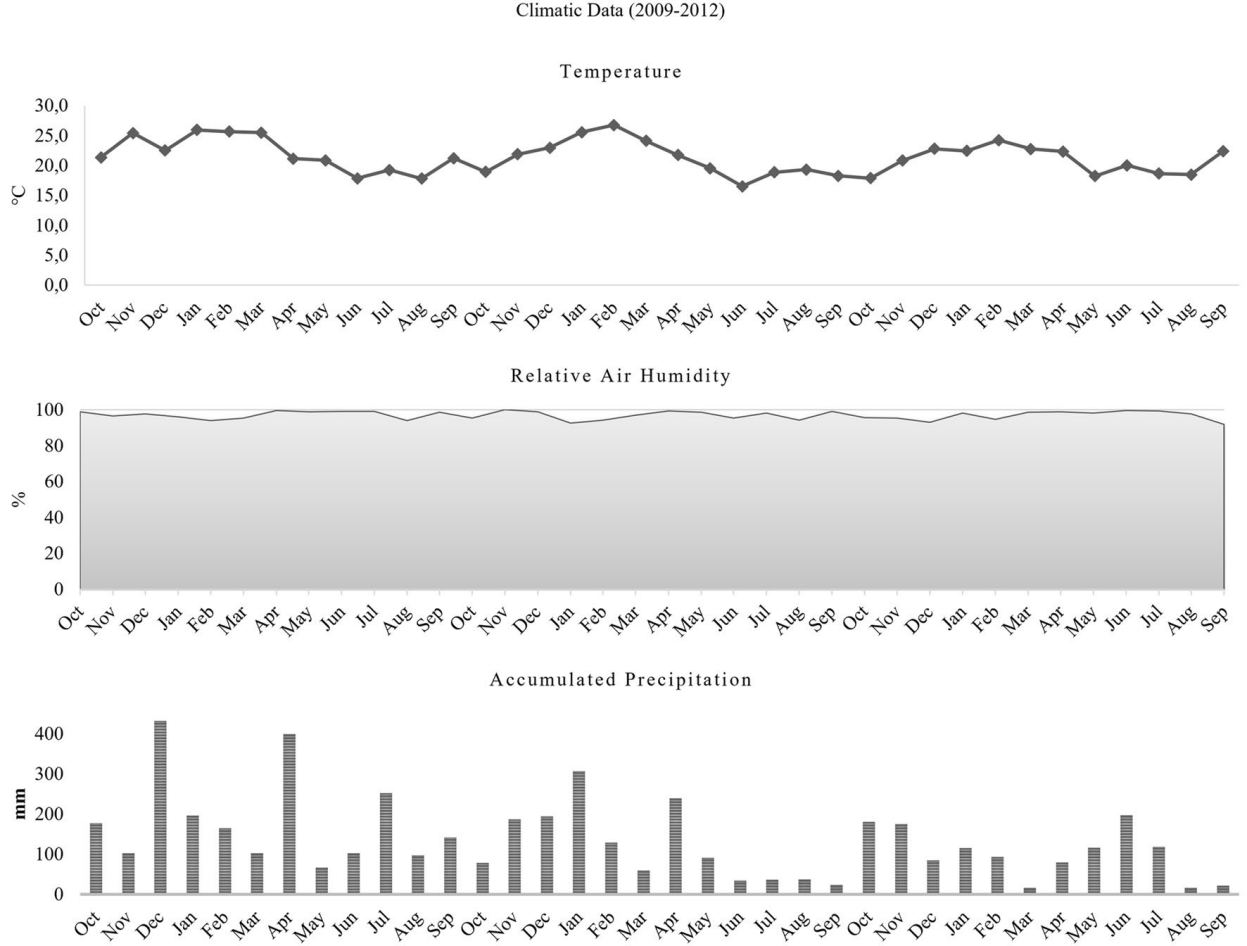
Climatic data from the National Institute of Meteorology of Temperature (°C), Relative Air Humidity (%) and Accumulated Precipitation, from October 2009 to September 2012, Municipality of Paraty, State of Rio de Janeiro, Brazil.

**Figure 3:**
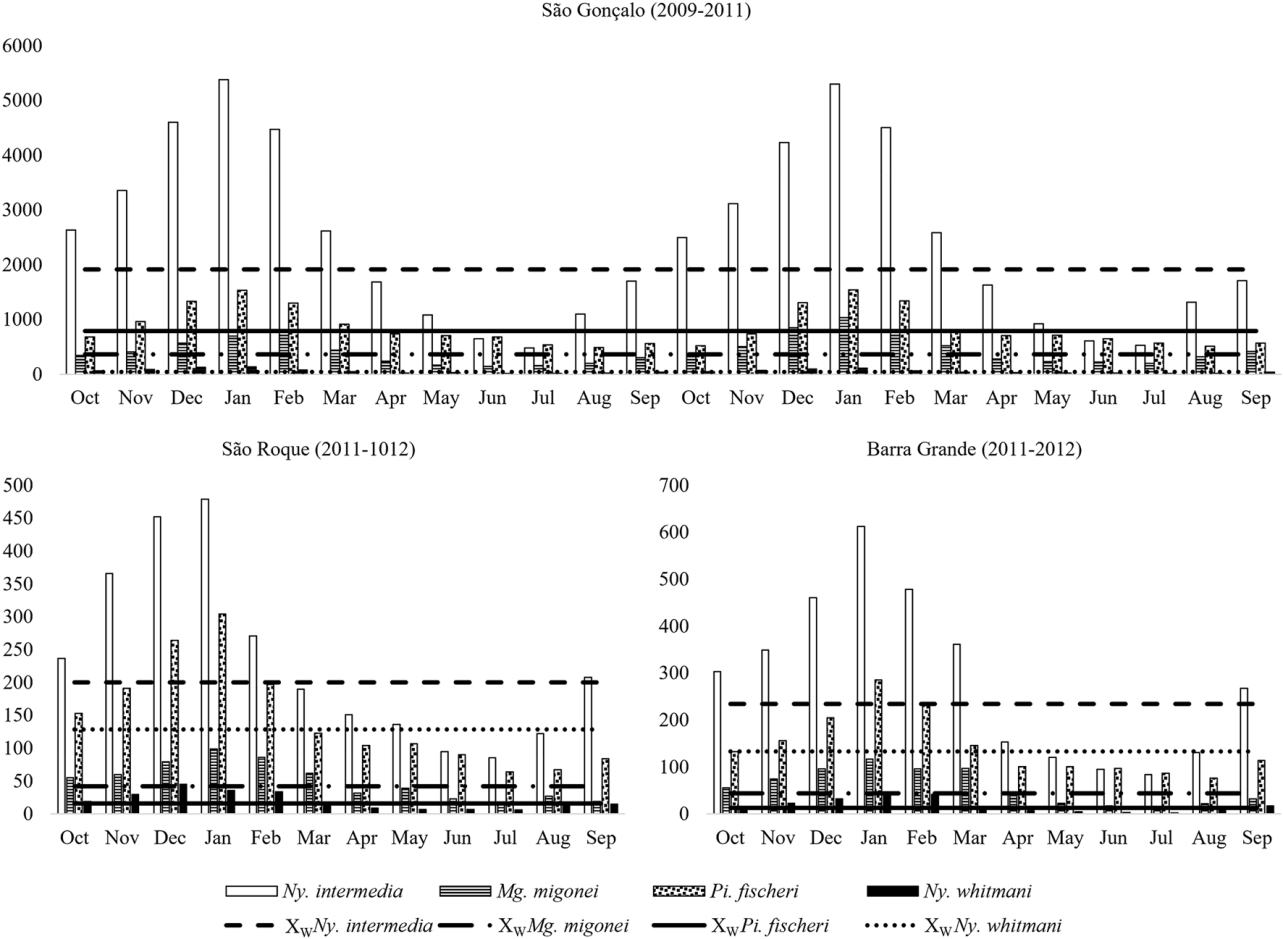
Monthly Frequency and Williams' Average (X_W_) of Sand flies: *Nyssomyia intermedia*, *Migonemyia migonei*, *Pintomyia fischeri* and *Nyssomyia whitmani*, from October 2009 to September 2012, in the neighborhoods of São Gonçalo, São Roque and Barra Grande, Municipality of Paraty, State of Rio de Janeiro, Brazil.

During the study period, there was high average relative air humidity in every month, above 90%. The months with the highest average rainfall in the first year was December, in the second, January and in the third year, June, with 432 mm, 307 mm 197 mm, respectively, and the lowest averages were in May (68 mm), in the first year, September (24 mm), on the second and March and August on the third (17 mm, each). The majority (more than 65%) of specimens were captured during the warmer months, between November and March, when temperature averages were close to 25 °C (maximum average of 27 °C and minimum of 21 °C), there was high relative humidity of above 93%, and high rainfall throughout the day (summer rains). In the cooler months, from June to August, when average temperatures were close to 18 °C (maximum temperatures averages 20 °C and 16 °C), relative humidity above 94%, and there was only moderate rainfall, the lowest frequency of local fauna was captured, accounting about only 10% of the specimens (Figs. 2 and 3).

Also in Fig. 3, the highest density of sand flies was observed in January in all three years of study, from 2009 to 2012, with 7,841 specimens, 8,080 and 2,016, respectively. In the neighborhood of São Gonçalo, São Roque and Barra Grande, the largest numbers of sand fly specimens were captured in the warmest months of the year from October to March. During these months, among the species of epidemiological importance, in the neighborhood of São Gonçalo, *Ny. intermedia* had the number of specimens captured higher than its Williams average of 1,916.8, in the two years; *Pi. fischeri* with the second highest average of 790.3 had numbers of specimens captured above this average from November to March in the first year and December to February in the second; *Mg. migonei* with an average of 363.1 from November to March in the first year and October to March in the second; and *Ny. whitmani* with averages of 39.9, in the months of October to March in the first year and October to February in the second year. Already in the district of Barra Grande, the number of sand flies of epidemiological importance captured above the Williams average were in the months of the year from October to March for *Ny. intermedia* with 234, *Pi. fischeri* with 133.4, *Mg. migonei* with an average of 44 and *Ny. whitmani* with 12.6; in the neighborhood of São Roque, the same period was observed for the species *Mg. migonei* with an average of 42 and *Ny. whitmani* with 16.1, and *Pi. fischeri* with an average of 128.7, had the species captured above average in the months of October to February and the same with *Ny. intermedia* with an average of 200, in addition to the month of September (Fig. 3).

Regarding Pearson's correlation, the numbers of captured sandflies had a strong and positive correlation (R = 0.7) in relation to temperature, that is, with the increase in temperature a greater number of sandflies were captured and in relation to relative air humidity (R = 0.2) and precipitation (R = 0.5) there was no correlation.

**Figure 4:**
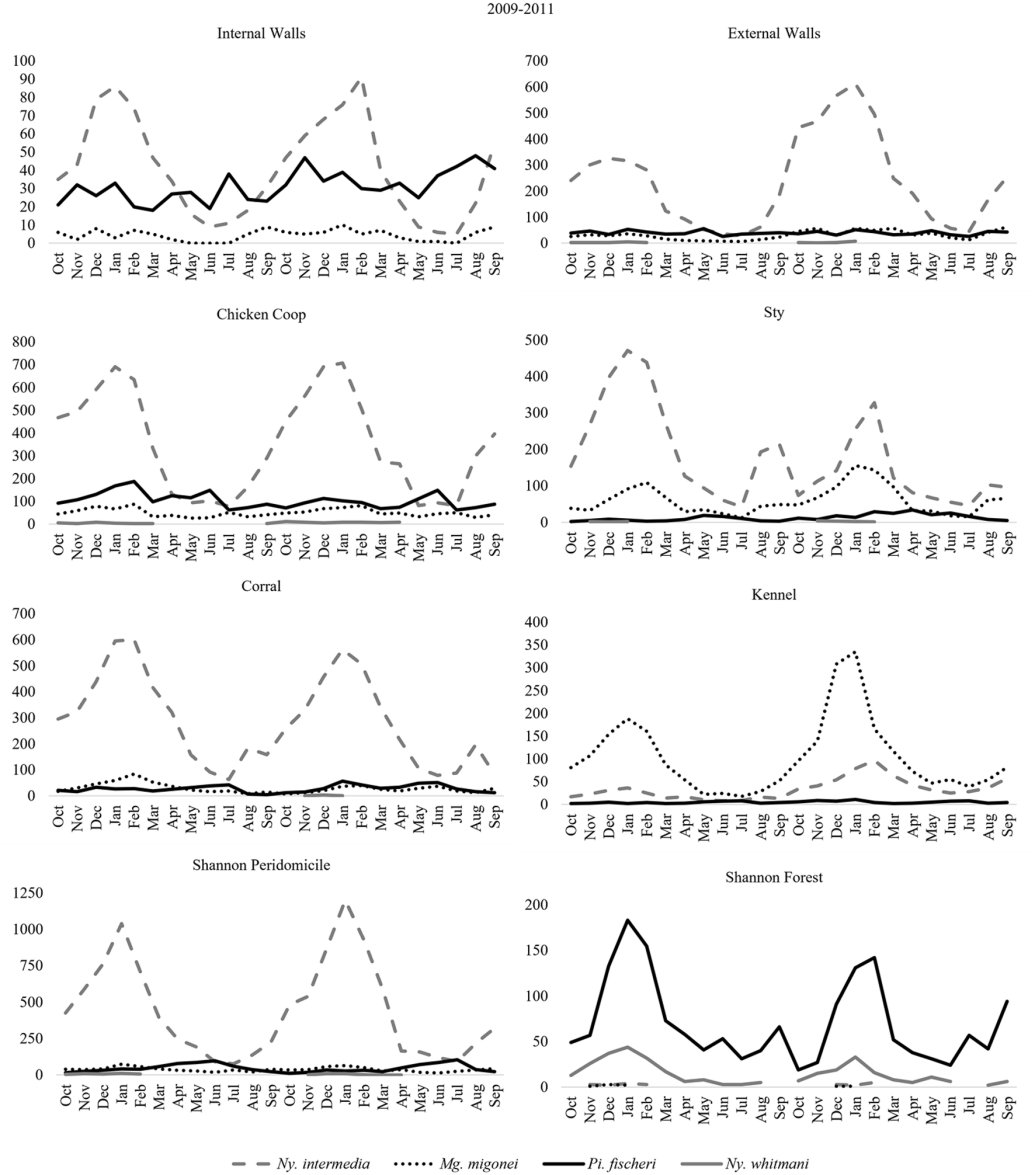
Monthly Frequency of Sand flies: *Nyssomyia intermedia*, *Migonemyia migonei*, *Pintomyia fischeri* and *Nyssomyia whitmani*, captured in the internal and external walls of the house, in the annex of domestic animals (chicken coop, sty, corral and kennel) and in the Shannon light trap (peridomicile and in the forest), from October 2009 to September 2011, in the neighborhoods of São Gonçalo, Municipality of Paraty, State of Rio de Janeiro, Brazil.

In the captures carried out on the internal and external walls of the house, it was observed that while *Ny. intermedia* had peak number of specimens in the warmer months; *Pi. fischeri* was present in more balanced numbers over the months of the year, surpassing the number of specimens of the most frequent species in the coldest months of the year in the internal walls and with balanced numbers on the external walls; *Mg. migonei* was present at the two capture sites; and *Ny*. *whitmani* was present in the captures made on the external walls. In the attachments of domestic animals while it is noted that *Ny. intermedia* and *Mg. migonei* are caught in greater numbers from the warmest months of the year; *Pi. fischeri* remains balanced over the months, with high numbers in both the hottest and coldest months of the year; in this location *Mg. migonei* draws attention to the number of specimens captured in the kennel, the only place where the species was more frequent in all months of the year; and *Ny*. *whitmani* it was not only present in the captures made in the kennel. In the captures with Shannon, it is observed that *Pi. fischeri* had a greater number of specimens captured in the coldest months of the year in the peridomicile, however in the forest, where the species was more frequent in all months of the year, the opposite was observed, with the largest number captured in the warmer months; *Ny*. *intermedia* and *Mg migonei* had little significant numbers captured in the forest, and only in the warmest months of the year; and *Ny*. *whitmani* was present both in the peridomestic environment and in the forest (Fig. 4).

**Figure 5:**
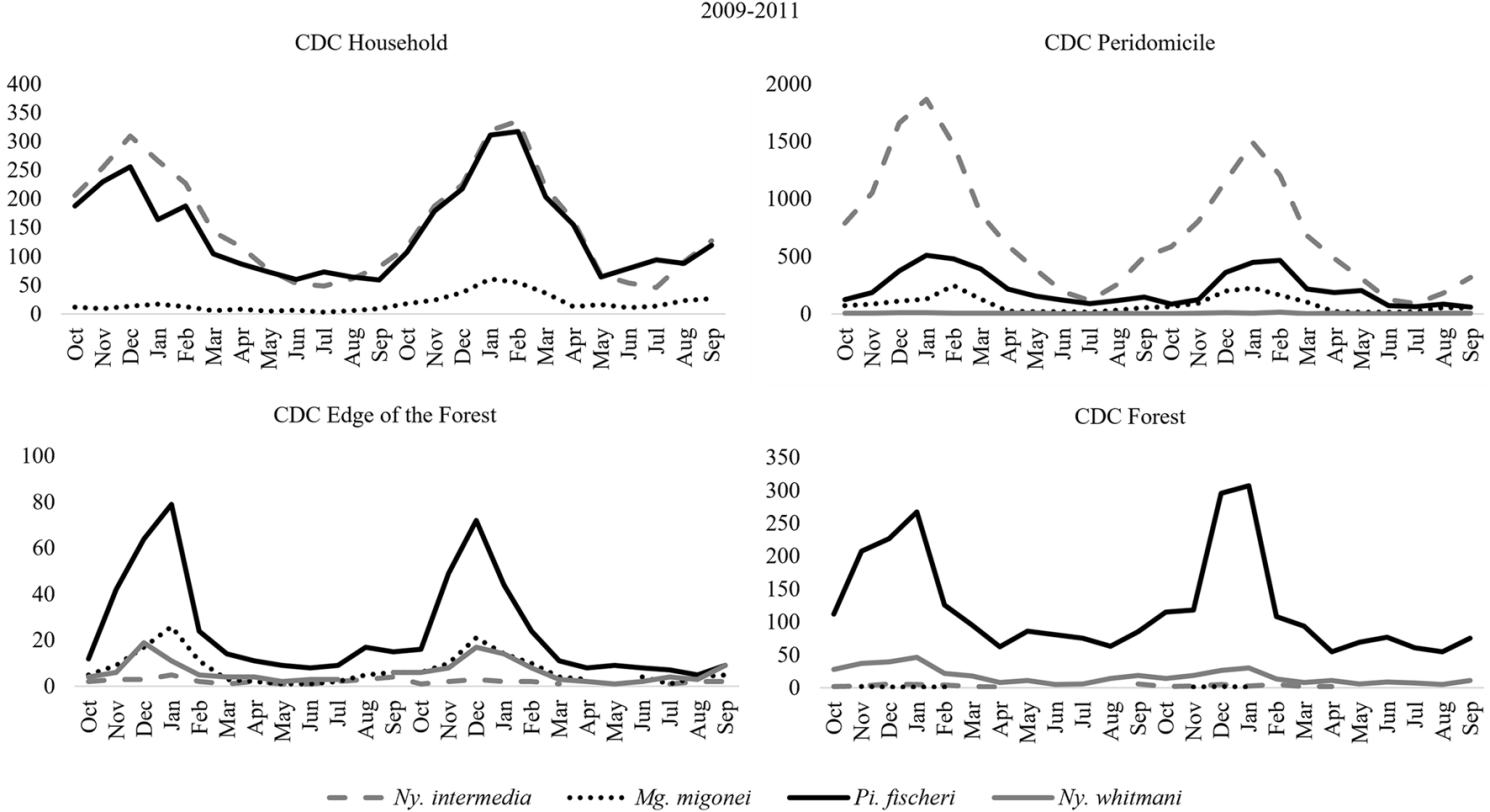
Monthly Frequency of Sand flies: *Nyssomyia intermedia*, *Migonemyia migonei*, *Pintomyia fischeri* and *Nyssomyia whitmani*, captured in the CDC light traps (household, peridomicile, edge of the forest and in the forest), from October 2009 to September 2011, in the neighborhoods of São Gonçalo, Municipality of Paraty, State of Rio de Janeiro, Brazil.

With the CDC light trap, in São Gonçalo, it was observed that *Ny. intermedia* and *Pi. fischeri* at home had balanced numbers throughout the year, with the highest number in the months of December to February and the same was observed in the peridomicile, including with *Mg. migonei*, however, *Ny. intermedia* had numbers much higher than the other two species throughout the year, except in the coldest months. Already both on the edge of the forest and in the forest, *Pi. fischeri* was the most frequent species in all months of the year, especially in the hottest periods, followed by *Mg. migonei* and *Ny. whitmani* who were in balanced numbers over the months in the forest margin and in the forest, at this location, for both *Mg*. *migonei* and *Ny. intermedia* had few specimens captured (Fig. 5).

**Figure 6:**
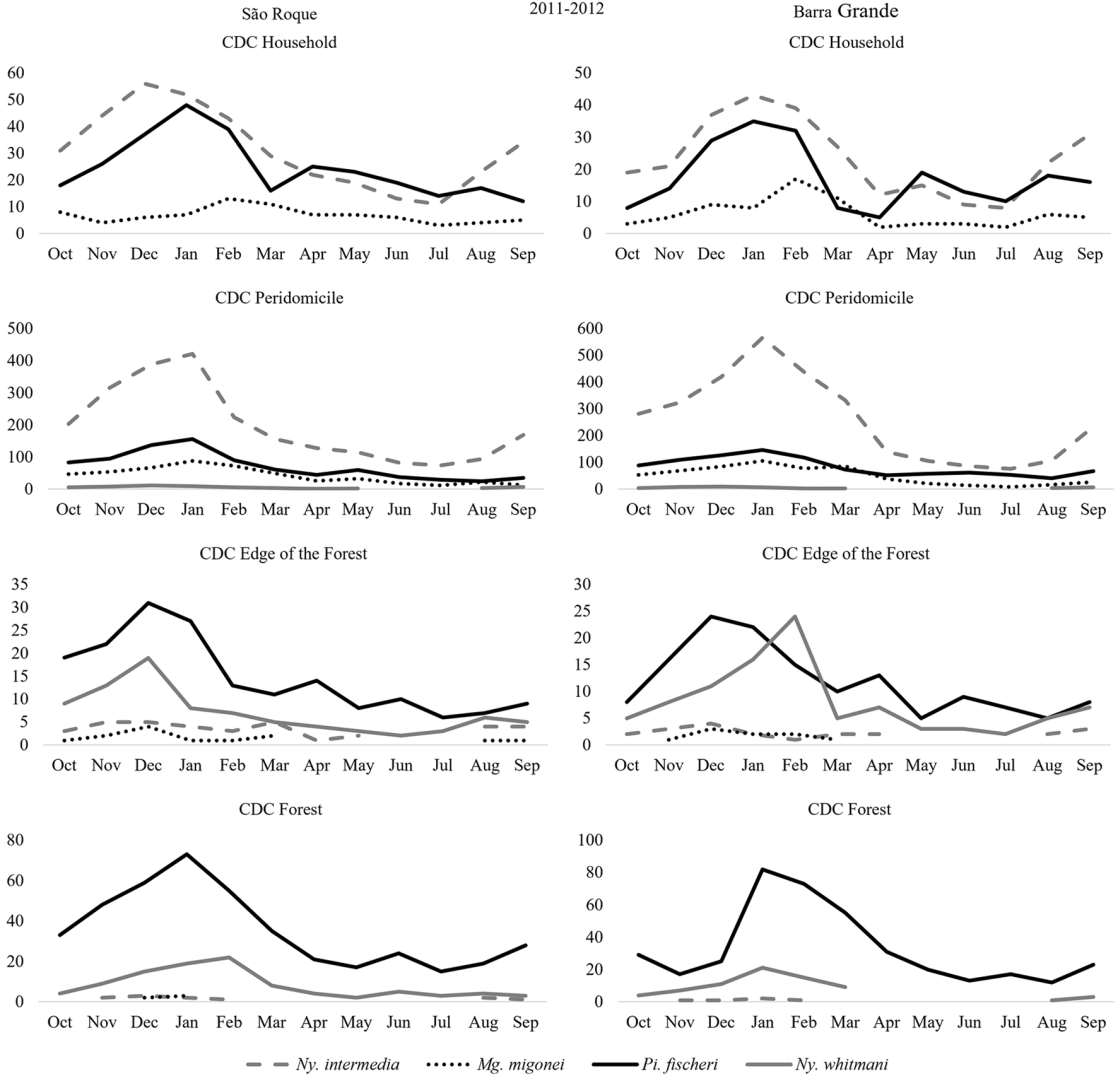
Monthly Frequency of Sand flies: *Nyssomyia intermedia*, *Migonemyia migonei*, *Pintomyia fischeri* and *Nyssomyia whitmani*, captured in the CDC light traps (household, peridomicile, edge of the forest and in the forest), from October 2011 to September 2012, in the neighborhoods of São Roque and Barra Grande, Municipality of Paraty, State of Rio de Janeiro, Brazil.

In the neighborhoods of São Roque and Barra Grande at home, *Ny. intermedia* and *Pi. fischeri* had close numbers of specimens captured over the months, however, from April to July in São Roque and May to July in Barra Grande, the second species surpassed the number of specimens captured from the first species. In the peridomicile *Ny. intermedia* was captured in supremacy over the months, except in the coldest months of the year, where the number of specimens is closer to the number of specimens captured of the species *Pi. fischeri* and *Mg. migonei*. On the edge of the forest *Pi. fischeri* and *Ny. whitmani* are the most frequent species in all months of the year and in Barra Grande in February this last species was captured in greater numbers than the first. In the forest *Pi. fischeri* was the only species present in significant numbers in all months of the year (Fig. 6).

## Discussion

Owing to drastic changes in the environment caused by human interference, wild mammals that are reservoirs of *Leishmania* have invaded residential areas where species of sand flies with eclectic feeding habits are found, and established a transmission cycle that eventually reaches humans^23–25^. In the study area, it was observed that the largest frequency of specimens over the years was captured in the residential environment, which are represented by residential and peridomicile areas. The lowest frequency was captured in the borders of the forest.

The municipality of Paraty, located on the southern coast in the state of Rio de Janeiro, where the study was conducted, has many preserved areas of the Atlantic Forest and its climate is wet with no dry season^13^, which was confirmed during the three years of the present study, where the relative air humidity stayed high every month. The highest average rainfalls occur in summer and fall (autumn). The average temperature during the hottest months of the year was between approximately 25 °C and 26 °C, with a maximum of 31 °C, and in the coldest months, the temperature averaged between 20 and 21 °C, with a minimum of 16 °C, exhibiting an ideal environment for the activity of sand flies throughout the year.

Barretto^26^ noted that atmospheric conditions, such as relative humidity, rainfall, and temperature directly influence the activity of these sand fly species. *Migonemyia migonei* and *Ny. whitmani* had lower activity at temperatures below 15 °C, *Pi. fischeri* below 10 °C, and *Ny. intermedia* at temperatures below 9.5 °C. The author also reported that heavy rains prevent sand flies from leaving their shelters; however, this can increase their density within residences, especially for species located next to residential areas. Light rain will not impede their activity, but in these conditions, they are not as frequently observed as they usually are. However, during rain periods, especially in the hot and humid summer period, the density of sand flies increases considerably.

In the present study, four key vector species of *Leishmania braziliensis* Vianna, 1911, the etiologic agent of tegumentary leishmaniasis, were captured throughout the year. The most frequent was *Ny. intermedia,* followed by *Pi. fischeri*, *Mg. migonei*, and *Ny. whitmani*. Carvalho et al.^27^, in the State of Pernambuco, northeast region of Brazil, reported having found *Mg. migonei* infected with *Leishmania infantum* Nicolle, 1908*,* the etiologic agent of visceral leishmaniasis.

According to Forattini^28^, there are sand fly species that are essentially resistant to climate changes throughout the seasons. Several are found, albeit in lower densities, during the cooler, dry months, while others disappear during this period. However, other factors also influence the incidence of sand flies in the same location, even under the same temperature and humidity conditions. Thus, to study the seasonality of sand fly species, it is important to perform systematized captures, for a period exceeding two years, to minimize the effects of these additional factors, for example, atypical years with a longer period of drought or humidity, more or less high temperatures, months with higher than expected rainfall or control measures applied by the municipality.

In studies carried out in the Northeast region of Brazil, in a study carried out in the municipality of Codó, in the State of Maranhão, an inversely proportional correlation of the captured sandflies was observed in relation to relative air humidity, a direct correlation in relation to temperature and precipitation, a correlation directly proportional^29^. In the municipality of Sobral, State of Ceará, in the first year of the study, observed a negative correlation with temperature and a high positive correlation with humidity and precipitation, however, in the following year, there was no correlation between the density of captured sandflies and climatic variables^30^. The same occurred in this study, in the municipality of Paraty, in relation to relative air humidity and precipitation, but in relation to temperature, a strong positive correlation was obtained.

In the studied area *Ny. intermedia* occurred in greater numbers in every month of the year, except in June and July, when it was less frequent than *Pi. fischeri*. The same pattern was observed for these two species, i.e., a gradual increase in abundance beginning in August, peak abundance in summer (January), followed by a decrease until winter (July). Brito et al.^31^, when researching the northern coast of the state of São Paulo, municipality of São Sebastião, noted the opposite, that *Ny. intermedia* had the highest abundance peaks during the driest and coldest period of the year, i.e., from May to August. However, the authors also emphasized the presence of this species throughout the year, mainly in the residential environment, and they stressed the importance of seasonal analyses for periods longer than a year.

In the São Francisco River region, in the state of Minas Gerais, on the banks of the Rio Velhas, Saraiva et al.^32^, in a study over a two-year period, observed a different pattern. In the first year of study, after the rainy season from February to May, with high humidity and high temperature, *Ny. intermedia* was captured in greater numbers than during other months of the year. In the second year, peaks occurred in October, March, and June, with the highest peak in March, when there was elevated rainfall, high humidity, and high temperatures.

In the state of Rio de Janeiro, in Serra dos Órgãos National Park, Aguiar and Soucasaux^33^ analyzed the monthly frequency in human bait and observed that *Ny. fischeri* was captured in every month except November. In the hot and humid period, from December to February, there was a gradual increase in the average abundances of this species, and then a slight decrease began in March and continued into April. During the cold and dry period of May and June, abundances started to increase, then decreased in July, and peaked in August. During August, *Pi. fischeri* was the dominant species of wildlife, and in September, abundances began to decline again.

Mayo et al.^34^, studying the southeastern region of the state of São Paulo, observed that there was a seasonal trend in the abundance for species *Mg. migonei*, *Ny. whitmani*, *Ny. intermedia*, and *Pi. fischeri,* with abundance peaks recorded during the cooler, drier season (April to September) and low abundances during the warmer, wetter season (October to March). The authors revealed that the occurrence of intense fires in the study area in October, which caused severe environmental change, possibly interfered with the population dynamics of the species. In the present study, the opposite trend of seasonality was shown for the four key species, *Ny. intermedia*, *Pi. fischeri*, *Mg. migonei*, and *Ny. whitmani,* then what was observed by the above authors, the highest abundances occurred during the hottest period, increasing gradually until a maximum peak in January, and lowest abundances were seen during the coldest period, in July for the first three species, and in June for *Ny. whitmani*.

In the neighboring municipality of this study in Angra dos Reis, in the Ilha Grande, Carvalho et al.^35^ reinforced the epidemiological importance of *Ny. intermedia* in the State of Rio de Janeiro and highlighted the role of *Mg. migonei* in the transmission of cutaneous leishmaniasis with its high rate of infection natural by *Leishmania*. Still in the same region, along the southern coast of the State of Rio de Janeiro, Aguiar et al.^8^ conducted systematic catches for two years, with the aim being to analyze the monthly frequency of sand flies in residential and forest environments. The authors discovered results like what occurred in this study in Paraty, that the four most important species caught, *Ny. intermedia*, *Pi. fischeri*, *Mg. migonei,* and *Ny. whitmani*, had higher average numbers during the hot and humid period of the year, i.e., between October and January, with a maximum peak in December for *Ny. intermedia* and *Pi. fischeri,* and January for *Mg. migonei.* The prevalence of *Ny. intermedia* was evident in every month, both inside the residence and around the residential area. In the colder and drier season, from May to August, there was a balance with *Pi. fischeri,* but from August, inside the residence, and from September, around the residence, the frequency increased until it reached its peak in December. There was a gradual increase in the frequency of this species in the warmer and wetter period (between October and January), with average temperatures ranging from 26 to 29 °C and relative air humidity between 84 and 87%.

Condino et al.^36^, when studying the southwestern region of the state of São Paulo, observed that *Ny. intermedia* and *Ny. whitmani* had the highest frequencies during the months of May, September, and December with temperatures ranging from 21 to 25.7 °C and rainfall between 66.7 and 195.1 mm. In June, the lowest frequency of sand flies was observed, which then increased until a maximum peak in September. Temperature data and rainfall index were not correlated with the density of specimens, especially as the study was carried out over only one year. In this study, the opposite was observed for *Ny. intermedia* and *Ny. whitmani* in the month of May, one of the months with the lowest density.

In the city of Petrópolis, state of Rio de Janeiro, Souza et al.^24^ observed a prevalence of *Ny. intermedia* and *Ny. whitmani,* with the latter species prevailing around the residence. *Migonemyia migonei* and *Pi. fischeri* were also present but to a lesser extent. In the forest, *Ny. whitmani* was more abundant, followed by *Pi. fischeri*, while *Ny. intermedia* was found at lower abundances. However, *Ny. intermedia* and *Pi. fischeri* were present during every month of the year. The authors also found a significant correlation between the number of sand flies and environmental changes such as temperature, relative humidity, and rainfall. The same was observed, in this study, in the forest with *Ny. intermedia*, however, in this environment the number of *Pi. fischeri* specimens was higher than that of *Ny. whitmani*.

In the north of Espírito Santo, Virgens et al.^37^ observed that *Ny. intermedia* was present in almost every month of the study period, with peaks in the warmer and wetter months. The authors highlighted that the low numbers of this species were recorded during and after high rainfall periods, suggesting that heavy rain is unfavorable for the development of immature forms, as breeding sites in altered habitats suffered a greater impact because of extreme weather conditions.

In a study carried out by Guimarães et al.^38^ to observe the competence of *Mg. Migonei* to *Leishmania infantum*, concluded that this species is highly susceptible to the development of this parasite and that in addition to its anthropophilia and abundance in areas with an active focus of visceral leishmaniasis, it can act as a vector of this disease in Latin America.

In the studied area, *Ny. intermedia*, one of the main vectors of the etiological agent of tegumentary leishmaniasis in the region^2^, was present in significant numbers in the home environment throughout all months of the year. The species *Pi. fischeri* was present over the months in expressive numbers in all types and locations of capture, that is, both in the environment altered by human activity and in the natural environment where leishmaniasis occurs in its natural enzootic cycle. *Migonemyia migonei*, present throughout the year in the peridomestic environment, showed its association with the dog, where it was prevalent throughout the year in the kennel, being an important vector of the etiological agent of tegumentary leishmaniasis, as well as being suspected in areas of visceral leishmaniasis transmission, where the main vector of this disease is not found. And *Ny. whitmani* present in the peridomicile, mainly in the hottest months of the year, in addition to the forest and forest margins, it was observed that in this study region the species is emerging through a selective process of adaptation in environments that were negatively affected by the increase of human activity. Thus, despite observing a period of greater frequency of sand flies in the hottest months of the year, a period with high rainfall, the high relative humidity is observed throughout the year, as well as the presence of species of epidemiological importance *Ny. intermedia*, *Pi. fischeri*, *Mg. migonei* and *Ny. whitmani*, who are involved in the propagation of the etiological agent of tegumentary leishmaniasis to humans and animals, causing greater contact between the region's inhabitants with these dipterans and thus, a greater risk of contracting the disease.
